# Ectodomain shedding of the hypoxia-induced carbonic anhydrase IX is a metalloprotease-dependent process regulated by TACE/ADAM17

**DOI:** 10.1038/sj.bjc.6602861

**Published:** 2005-11-08

**Authors:** M Zatovicova, O Sedlakova, E Svastova, A Ohradanova, F Ciampor, J Arribas, J Pastorek, S Pastorekova

**Affiliations:** 1 Center of Molecular Medicine, Institute of Virology, Slovak Academy of Sciences, Dubravska cesta 9, Bratislava 845 05, Slovak Republic; 2 Medical Oncology Research Program, Vall d'Hebron University Hospital Research Institute, Barcelona 08035, Spain

**Keywords:** carbonic anhydrase IX, hypoxia, ectodomain shedding, metalloproteases, TACE/ADAM17

## Abstract

Carbonic anhydrase IX (CA IX) is a transmembrane protein whose expression is strongly induced by hypoxia in a broad spectrum of human tumours. It is a highly active enzyme functionally involved in both pH control and cell adhesion. Its presence in tumours usually indicates poor prognosis. Ectodomain of CA IX is detectable in the culture medium and body fluids of cancer patients, but the mechanism of its shedding has not been thoroughly investigated. Here, we analysed several cell lines with natural and ectopic expression of CA IX to show that its ectodomain release is sensitive to metalloprotease inhibitor batimastat (BB-94) and that hypoxia maintains the normal rate of basal shedding, thus leading to concomitant increase in cell-associated and extracellular CA IX levels. Using CHO-M2 cells defective in shedding, we demonstrated that the basal CA IX ectodomain release does not require a functional TNF*α*-converting enzyme (TACE/ADAM17), whereas the activation of CA IX shedding by both phorbol-12-myristate-13-acetate and pervanadate is TACE-dependent. Our results suggest that the cleavage of CA IX ectodomain is a regulated process that responds to physiological factors and signal transduction stimuli and may therefore contribute to adaptive changes in the protein composition of tumour cells and their microenvironment.

Carbonic anhydrases (CAs) are zinc metalloenzymes that catalyse a reversible conversion of carbon dioxide to bicarbonate and proton in a reaction: CO_2_+H_2_O↔HCO_3_^−^+H^+^. This simple reaction is fundamental to physiological processes based on gas exchange, ion transport and pH balance, such as respiration, production of body fluids, digestion, bone resorption, renal acidification, etc. Therefore, CAs are broadly distributed in various living organisms and throughout the body in different tissues, cell types and subcellular compartments (Pastorekova *et al*, 2004). Human CAs exist in at least 15 isoforms, which involve 12 active isoenzymes: cytoplasmic CA I, II, III, VII, XIII, mitochondrial CA VA and VB, secreted CA VI and membrane-bound CA IV, IX, XII and XIV. The isoenzymes exhibit variable catalytic activity, kinetic properties and sensitivity to sulphonamide inhibitors (Supuran, 2004). Carbonic anhydrase IX (CA IX, initially named MN or G250) is a highly active and sulphonamide-avid isoform (Wingo *et al*, 2001; Vullo *et al*, 2003). In contrast to other CA isoenzymes, CA IX is normally present in only few normal tissues, namely in epithelia of the gastrointestinal tract, whereas it is abnormally induced in various human tumours including carcinomas of the kidney, lung, breast, colon, oesophagus, breast, uterine cervix, etc. (Pastorekova and Zavada, 2004). This tumour-associated distribution of CA IX is related to a strong transcriptional activation of the *CA9* gene by a hypoxia-inducible factor 1, a key mediator of the molecular responses to low oxygen supply (Wykoff *et al*, 2000). Accordingly, CA IX is predominantly found in the perinecrotic hypoxic regions and signifies poor prognosis (Potter and Harris, 2003). However, CA IX is not just a surrogate marker of tumour hypoxia, but appears to be functionally implicated in tumorigenesis via its capacity to modulate cell adhesion and acidify the microenvironment of hypoxic cells (Svastova *et al*, 2003, 2004).

Carbonic anhydrase IX is a type I membrane protein of 58/54 kDa. Its extracellular portion contains a large CA domain, which is N-terminally extended with a proteoglycan (PG)-like region, and via a single-pass transmembrane (TM) region is linked to a short C-terminal intracytoplasmic (IC) tail (Pastorek *et al*, 1994; Opavsky *et al*, 1996). The extracellular domain (ECD) of CA IX can be released into the cell culture medium or into the body fluids of tumour patients (Zavada *et al*, 2003). Concentration of CA IX ECD in the blood and urine varies in a wide range and shows no obvious correlation with the tumour size, at least in patients with renal cell carcinomas. On the other hand, CA IX ECD is cleared from the blood within a few days after nephrectomy and its concentration in control individuals is extremely low (Zavada *et al*, 2003). Thus, it has been proposed that the detection of CA IX ECD in the body fluids can find a clinical application in screening and monitoring of tumour patients.

Recent evidence indicates that the ectodomain shedding is a biologically relevant phenomenon, which affects the fate, location, level and function of certain cytokines, growth factors, receptors, cell-adhesion molecules, ectoenzymes, etc. It appears to occur at or near the cell surface, generally depends upon the action of matrix metalloproteinases (MMP) or adamalysins (ADAMs, for a disintegrin and metalloproteinase) and can be often induced by phorbol ester activators of protein kinase C (Arribas *et al*, 1996; Werb and Yan, 1998; Schlondorff and Blobel, 1999).

Molecular mechanisms that underlie the release of CA IX ECD have not been examined so far. Here, we analysed several cell lines with natural and ectopic expression of CA IX for their capacity to shed CA IX. We found that the CA IX ECD release is sensitive to metalloproteinase inhibitor BB-94 and that hypoxia can retain a normal rate of shedding producing more ECD concomitantly with augmented expression of the protein in the cell. We also used CHO cells, their shedding-defective mutant M2 cells and TACE-transfected M2 cells to demonstrate that the constitutive CA IX ectodomain release does not require functional TNF*α*-converting enzyme (TACE/ADAM17), whereas induction of CA IX ECD shedding by both phorbol-12-myristate-13-acetate and pervanadate is TACE-dependent. Our results suggest that production of CA IX ECD is a metalloprotease-mediated process that responds to different signals and may therefore participate in adaptive changes in the protein composition of tumour cells and of their microenvironment.

## MATERIALS AND METHODS

### Cell lines and culture conditions

Tumour cell lines HeLa, SiHa, Caski (derived from cervical carcinoma), HT-29 (colon carcinoma), ACHN (renal cell carcinoma), CGL1 and CGL3 (hybrids between HeLa and normal fibroblasts; Stanbridge *et al*, 1981) were grown in DMEM (BioWhittaker, Verviers, Belgium) supplemented with 10% FCS, at 37°C in air with 5% CO_2_. The same culture conditions were applied to MDCK canine kidney cells permanently transfected with the full-length *CA9* cDNA in pSG5C plasmid (MDCK+CA IX; Svastova *et al*, 2003), to permanently transfected C33a+CA IX cervical carcinoma cells, to Ka13 derivative of CHO-K1 Chinese hamster cells lacking HIF-1*α* protein (kindly provided by Dr Patrick Maxwell, Imperial College of Science, Technology and Medicine, London, UK; Wood *et al*, 1998) and to CHO-WT, CHO-M2, CHO-M2+TACE cells with normal, defective and overexpressed TACE/ADAM17, respectively (Villanueva de la Torre *et al*, 2004). The cells were exposed to hypoxia (2% O_2_) in an anaerobic workstation (Ruskin Technologies, Bridgend, UK) with 5% CO_2_, 10% H_2_ and 83% N_2_ at 37°C. Transient transfection of CHO-derived cell lines with the pSG5C-CA9 plasmid containing the full-length *CA9* cDNA (Pastorek *et al*, 1994) was performed using the Gene Porter II transfection reagent according to the instructions of the manufacturer (Gene Therapy Systems, San Diego, CA, USA).

### Antibodies and chemicals

M75 mouse monoclonal antibody (MAb) specific for the N-terminal PG region of the CA IX protein and V/10 mouse MAb specific for the CA domain of CA IX were described previously (Pastorekova *et al*, 1992; Zatovicova *et al*, 2003). Purified M75 was labelled with NHS-LC-Biotin (Pierce, Rockford, IL, USA) according to the instructions of the manufacturer. Briefly, 2 mg of purified IgG dissolved in PBS, pH 8.0, were incubated with 100 *μ*g NHS-LC-Biotin for 2 h on ice. Free biotin was removed using microconcentrator (PALL Gelman Lab., Wien, Austria) or gel filtration. Secondary anti-mouse antibodies conjugated with horseradish peroxidase were purchased from Sevapharma (Prague, Czech Republic) and peroxidase-conjugated streptavidin was obtained from Pierce (Rockford, IL).

Metalloprotease inhibitor BB-94 (batimastat; British Biotechnology Ltd, Oxford, UK) and phorbol-12-myristate-13-acetate (PMA; Sigma, St. Louis, MO) were dissolved in dimethyl sulphoxide (DMSO) at 10 mM, and stored in aliquots at −20°C. Pervanadate was prepared by mixing equimolar concentrations of orthovanadate (Sigma) and H_2_O_2_ just before the experiment. Prior to use, the inhibitors were diluted in culture medium to working concentrations (1–10 *μ*M BB-94, 10 *μ*M PMA and 200 *μ*M pervanadate). Recombinant epidermal growth factor (EGF; Austral Biologicals, San Ramon, CA, USA) was used at 100 ng ml^−1^ final concentration.

### Biotinylation, immunoprecipitation and immunoblotting

Cells in monolayers of 70–80% confluence grown in a 6 cm dish were washed with ice-cold buffer A (20 mM sodium hydrogen carbonate, 0.15 M NaCl, pH 8.0). Immediately before use, 1 mg of NHS-LC-Biotin (Pierce) was dissolved in 50 *μ*l DMSO, mixed with 4 ml buffer A, added to cells and incubated for 60 min at 4°C. The cells were then washed five times with buffer A and after an indicated time period solubilised in ice-cold RIPA buffer (1% Triton X-100 and 1% deoxycholate in PBS) containing the COMPLETE cocktail of protease inhibitors (Roche Diagnostics GmbH, Mannheim, Germany) for 30 min on ice. The extracts were collected, cleared by centrifugation at 15 000 r.p.m. for 10 min at 4°C and stored at −80°C. Protein concentrations in extracts were quantified using the BCA protein assay reagent (Pierce, Rockford, IL, USA).

For immunoprecipitation, MAb V/10 in a volume of 1 ml culture medium was bound to 25 *μ*l 50% suspension of Protein-A Sepharose (Pharmacia, Uppsala, Sweden) for 2 h at room temperature. Biotinylated cell extract (200 *μ*l) or medium collected from the cell culture (2 ml) was precleared with 20 *μ*l of 50% suspension of Protein-A Sepharose and then added to bound MAb. Immunocomplexes collected on Protein-A Sepharose were washed and separated by SDS–polyacrylamide gel (PAGE) gel (10%) electrophoresis as described before (Zatovicova *et al*, 2003). Afterwards, the proteins were transferred onto PVDF membrane, revealed either with peroxidase-conjugated streptavidin (1 : 1000; Pierce) or with biotin-labelled M75 followed by peroxidase-conjugated streptavidin and visualised by enhanced chemiluminescence.

### ELISA

The capture antibody V/10 (10 *μ*g ml^−1^) was immobilised on the surface of microplate wells overnight at 4°C. After blocking and washing, the cell extracts or media diluted in PBS were added to the coated wells for an overnight binding at 4°C. The attached antigen was then allowed to react with biotinylated MAb M75 diluted 1 : 5000 (200 ng ml^−1^) in PBS. The amount of bound detector antibody was determined after 1 h incubation with the peroxidase-conjugated streptavidin (Pierce) using a peroxidase substrate orthophenylene diamine (Sigma).

### Immunofluorescence

Cells grown on glass coverslips were washed and kept in Ca^2+^-free PBS for 20 min to allow for their detachment. The remaining cells were fixed in ice-cold methanol at −20°C for 5 min. Nonspecific binding was blocked by incubation with PBS containing 1% BSA for 30 min at 37°C. Then, the cells were incubated with M75 antibody for 1 h at 37°C followed by anti-mouse FITC-conjugated horse antibody (Vector Laboratories, Burlingame, CA, USA) diluted 1 : 300 in PBS–BSA for 1 h at 37°C. The nuclei were stained with propidium iodide (Sigma). Finally, the coverslips were mounted onto slides in a mounting medium with Citifluor (Agar Scientific Ltd, Essex, UK) and analysed by a confocal laser-scanning microscope Fluoview Olympus IX 70. Images were generated using Fluoview personal confocal microscope system version 1.2 software.

### RT–PCR

Total RNA was extracted from cells using NucleoSpin RNA II kit (Macherey-Nagel, Düren, Germany). Precipitated RNA was dissolved in DEPC-treated water and reverse transcribed with the M-MuLV reverse transcriptase (Finnzymes, Oy, Finland) using random hexanucleotides as primers. PCR reactions were performed using the primers listed in Table 1 and EXT DNA polymerase (Finnzymes, Oy, Finland). Following an initial denaturation at 95°C for 3 min, the amplification programme was set as follows: denaturation at 95°C for 30 s, annealing at 58–60°C for 40 s and extension at 72°C during 40 s for a total of 30 cycles, and finally 5 min at 72°C. The RT–PCR products were analysed on a 2% agarose gel.

### Zymography

Culture media were collected from the cells grown for 24 h in confluent monolayers, centrifuged (5000 r.p.m. for 5 min at 4°C) to remove cell debris and applied to a nonreducing SDS–PAGE containing 0.1% collagen isolated from the rat tail. The electrophoresed gels were washed to remove SDS, renatured for 1 h in 2.5% Triton X-100 at room temperature and incubated for 40 h at 37°C in the substrate buffer (50 mM Tris-base, 0.2 M NaCl, 5 mM CaCl_2_, 1 *μ*M ZnCl_2_, pH 7.6) to allow for the enzymes digesting the collagen. After staining with 0.5% CBB, the gels were dried and photographed.

## RESULTS

### Rate of basal CA IX ECD release

**Figure 1:**
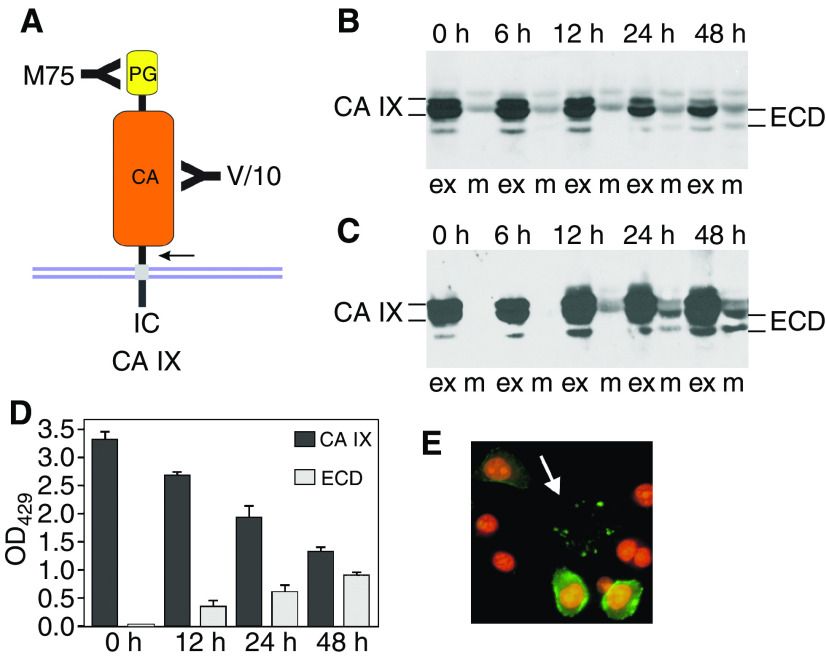
Basal carbonic anhydrase IX (CA IX) ectodomain shedding from CGL3 cells. (**A**) Schematic illustration of CA IX molecule with an extracellular portion composed of N-terminal proteoglycan (PG)-like region and central carbonic anhydrase (CA) domain linked via a single transmembrane segment to a short intracytoplasmic (IC) tail. Detection of the ectodomain was enabled by two noncompetitive CA IX-specific monoclonal antibodies: V/10 directed to CA and M75 directed to PG. The arrow indicates a putative cleavage site whose exact position is unknown. (**B**) CA IX ECD release was analysed by immunoprecipitation–immunoblotting analysis of the aliquots of nondiluted extracts (ex) and media (m) corresponding to the same number of cells obtained from the biotinylated CGL3 cultures that were chased for various time periods. CA IX was first precipitated with V/10 MAb, blotted and revealed with streptavidin–peroxidase. (**C**) The same blot was stripped and reprobed with the biotinylated M75 followed by streptavidin–peroxidase. (**D**) CA IX ECD release from biotinylated CGL3 cells was assessed also by sandwich ELISA using V/10 antibody as a capture and streptavidin–peroxidase as a detector. For this purpose, cell extracts were diluted 20 times, whereas media were diluted 1:2. OD_492_ values represent average levels of CA IX measured in triplicates in at least three independent experiments, and standard deviations are indicated. (**E**) CGL3 cells grown on glass coverslips were allowed to detach in Ca^2+^-free PBS and then subjected to immunofluorescence labelling with M75 and FITC-conjugated anti-mouse antibodies. The cell nuclei were stained with propidium iodide. The arrow points to CA IX-positive ‘traces’ left after the detachment of CGL3 cell.

To analyse the rate of a basal shedding of CA IX ectodomain, we used CGL3 hybrid cell line generated by fusion of HeLa cervical carcinoma cells with normal human fibroblasts, which expresses relatively high level of CA IX under standard cultivation conditions (Zavada *et al*, 1993). The CGL3 cells grown in a confluent monolayer for 24 h were first labelled by biotinylation and then chased for different time periods throughout the subsequent 48 h. Level of CA IX present in the cellular extract and in the corresponding culture medium was assessed by immunodetection with two noncompetitive CA IX-specific MAbs directed to distinct extracellular domains: M75 MAb against an N-terminal PG-like domain and V/10 MAb against a juxtamembrane CA domain (Figure 1A). Carbonic anhydrase IX was immunoprecipitated with V/10 MAb and blotted onto the membrane. The membrane was then treated either with the streptavidin–peroxidase to visualise only the biotin-labelled fraction of the protein actually expressed at the beginning of the shedding period (Figure 1B) or with biotin-labelled M75 followed by streptavidin–peroxidase to visualise also the protein synthesised *de novo* and shed during the chase (Figure 1C). Both approaches showed that the proportion of the released CA IX ECD compared to the cell-bound protein is rather low and the process of basal shedding is relatively slow. Molecular weight of the CA IX ECD was decreased by about 4 kDa compared to the full-length CA IX protein and this reduction corresponded roughly with the calculated mass of the removed TM and IC regions. The basal shedding of CA IX ECD was also analysed by ELISA, based on the same principle as the immunoprecipitation-immunoblotting experiment described on Figure 1B. Resulting absorbance values were in accord with the levels of CA IX seen on the blot and showed that CA IX accumulation in the medium is accompanied by a reduced amount of the cell-bound protein (Figure 1D). Interestingly, CA IX ECD was detectable not only in the medium, but it was regularly observed on the coverslips after detachment of nonfixed, viable CA IX-positive cells before their immunofluorescence labelling with anti-CA IX antibodies. Carbonic anhydrase IX-specific signal in the form of the ‘cell traces’ was clearly visible in CGL3 culture in the absence of the cell body including nucleus (Figure 1E). This finding indicated that CA IX was shed from the entire surface of tumour cells including the ventral side contacting the solid support.

### Shedding of CA IX ECD in various cell lines under normoxia and hypoxia

**Figure 2:**
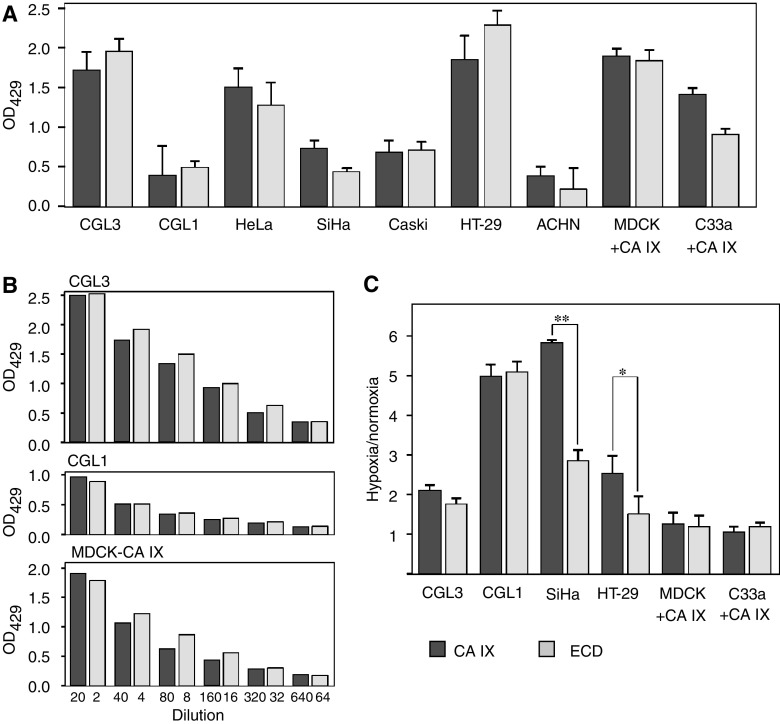
Basal rate of carbonic anhydrase IX (CA IX) ectodomain release from various cell lines under normoxia and hypoxia. (**A**) Confluent monolayers of CA IX-expressing cells were washed, fresh medium was added and the shedding was allowed to proceed over a 48-h period. The media were collected, the cells were extracted and the content of CA IX was determined by ELISA with V/10 as a capture and biotinylated M75 followed by streptavidin–peroxidase as a detector. (**B**) Serial dilutions of the extracts and media were examined by ELISA to relate the cellular and extracellular CA IX concentrations. (**C**) The parallel cultures were maintained for 48 h in normoxia and hypoxia (2% O_2_). Harvested media (diluted 1:2) and cell extracts (diluted 1:20) were assayed in ELISA and the hypoxia-induced changes of CA IX levels in the cells and released to medium were illustrated as a ratio between the absorbance values measured in the hypoxic samples and the values of the corresponding normoxic samples. ^*^P<0.01 and ^**^P<0.001 by Student's *t*-test.

The level of CA IX expression considerably varies in different cell lines, thus raising a question whether the basal ectodomain shedding reflects abundance of the CA IX protein in the cells or whether there are any cell-type-related variations. To answer this question, we examined several tumour cell lines that naturally express CA IX and two transfected cell lines with a constitutive ectopic CA IX expression (MDCK+CA IX and C33a+CA IX) for their capacity to shed CA IX ECD. The cells were grown in a confluent monolayer and ECD release was evaluated after a shedding period of 48 h. Extracts and media were added to V/10 MAb-coated wells and bound CA IX was detected with biotin-labelled M75 MAb followed by streptavidin–peroxidase. As shown in Figure 2A, ECD levels generally corresponded to levels of the cell-bound CA IX protein, so that the cells with a high CA IX expression such as CGL3, HeLa, HT-29 and both transfectants displayed relatively high levels of ECD and the cells with a low CA IX expression such as CGL1 and ACHN released correspondingly less ECD. The production of CA IX ECD was slightly lower in SiHa cells and in C33a+CA IX transfectants when compared to the other examined cell lines. More thorough assessment of the quantitative relationships between the cellular and shed CA IX in ELISA with titration of the extracts and media from the cells with high, low and constitutive CA IX expression, respectively, showed that the proportion of the released CA IX ECD is close to 10% in all three cases (Figure 2B). This finding supports the idea that the basal CA IX shedding is a steady process that operates in different cells to cleave off an approximately same fraction of the CA IX molecules.

Expression of CA IX is strongly induced by hypoxia in an HIF-1-dependent manner (Wykoff *et al*, 2000). This induction results in a highly increased abundance of CA IX protein in the hypoxic cells both in culture and in tumour tissues (Potter and Harris, 2003). In addition, hypoxia can improve a catalytic capacity of CA IX to acidify extracellular pH (Svastova *et al*, 2004). However, it is not known whether and how hypoxia influences the shedding of CA IX ECD. Therefore, we measured the amounts of the cellular CA IX and ECD in extracts and media from the cells exposed to hypoxia (2% O_2_) for 48 h. Hypoxic treatment of CGL3 and CGL1 cells led to increased cellular levels of CA IX accompanied by accordingly elevated production of an extracellular CA IX ECD, whereas ECD accumulation was lower in SiHa and HT-29 cells compared to their cellular CA IX (Figure 2C). Nevertheless, hypoxic shedding of CA IX ECD in the transfected cells that constitutively express CA IX from SV40 promoter remained similar to shedding under normoxic conditions, suggesting that the rate of the basal CA IX shedding was principally not affected by hypoxia.

### Inhibition of basal CA IX ECD shedding by BB-94

**Figure 3:**
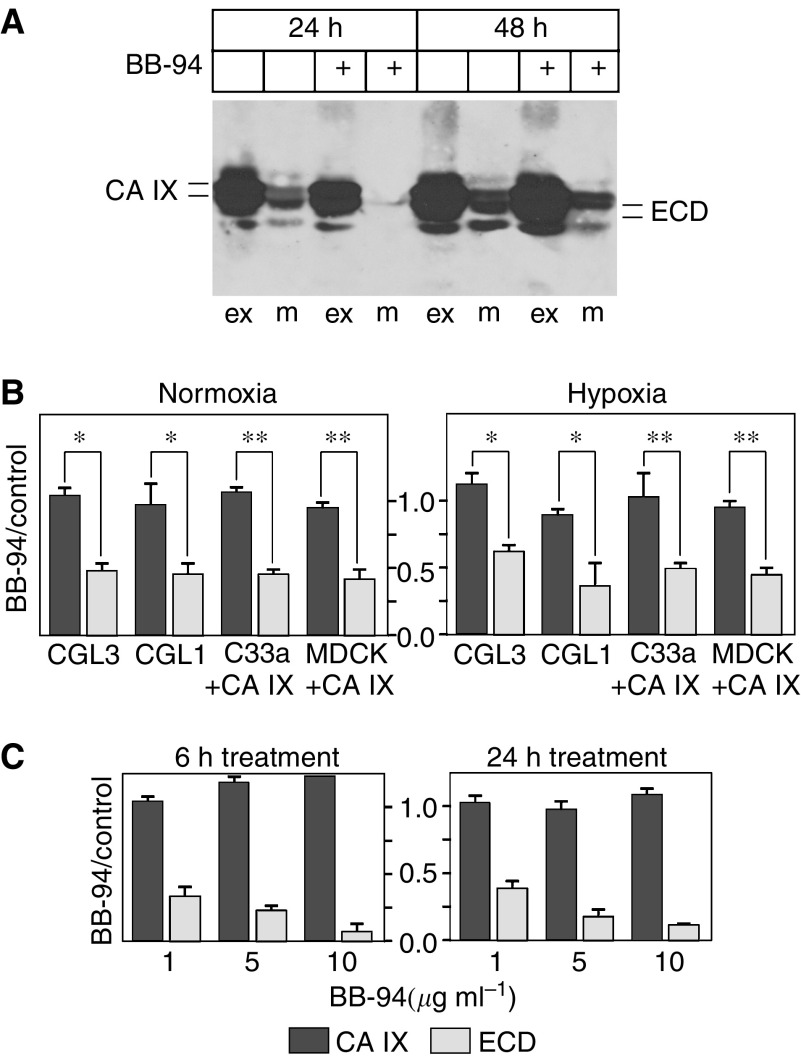
Inhibition of the basal carbonic anhydrase IX (CA IX) shedding with BB-94 metalloprotease inhibitor. (**A**) CGL3 cells were biotinylated and then allowed to shed CA IX ECD in the presence and absence of 1 *μ*M BB-94. The media (m) were collected, the cells were extracted (ex) and the corresponding aliquots of the materials were analysed by immunoprecipitation–immunoblotting as described in Figure 1c. (**B**) ELISA was used to determine the inhibition of CA IX shedding with 1 *μ*M BB-94. The cells were treated for 24 h throughout their incubation in normoxia or hypoxia. Extent of inhibition was illustrated as a ratio of the absorbance values of the materials from BB-94-treated cells *vs* their nontreated counterparts normalised according to total protein concentrations. (**C**) ELISA assessment of a BB-94 concentration-dependent inhibition of CA IX ECD release in MDCK+CA IX cells. The results are expressed as a ratio between the BB-94-inhibited and control samples. ^*^P<0.01 and ^**^P<0.001 by Student's *t*-test.

Numerous cell-surface proteins are shed by a mechanism that involves metalloproteases, particularly the matrix metalloproteases and members of the ADAM family of proteins. The activity of these proteases is sensitive to BB-94 inhibitor (batimastat). Therefore, we used this inhibitor to treat the cells and see whether it can influence the shedding of CA IX. Immunoblotting of CA IX protein immunoprecipitated from CGL3 cells grown in the presence of BB-94 revealed a considerably reduced production of the CA IX ECD (Figure 3A). This finding was confirmed by sandwich ELISA, which showed that 1 *μ*M BB-94 inhibited CA IX ECD shedding to about half of the control value. Similar reduction was observed in other three cell lines, namely CGL1 and transfected C33a+CA IX as well as MDCK+CA IX cells, and appeared comparable under hypoxia (Figure 3B). Moreover, ECD shedding was inhibited by BB-94 in a concentration-dependent manner in CA IX-transfected MDCK cells where 10 *μ*M BB-94 decreased the level of ECD to less than a tenth of a control value (Figure 3C).

### Regulated shedding of CA IX ECD

**Figure 4:**
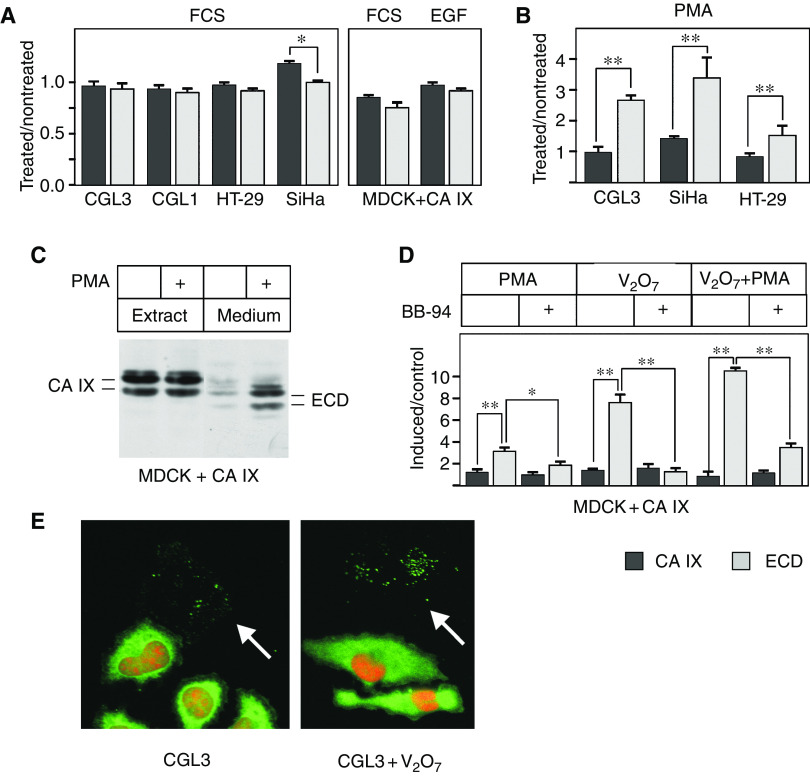
Activation of carbonic anhydrase IX (CA IX) ectodomain release by phorbol-12-myristate-13-acetate (PMA) and pervanadate. (**A**) The cells were first serum starved and then incubated with fresh medium in the presence or absence of 10% FCS, or alternatively in the presence or absence of epidermal growth factor (EGF), for 24 h. The extracts and media were analysed by ELISA and the results were illustrated as a ratio between the stimulated and nonstimulated cells. (**B**) The serum-starved cells were allowed to release CA IX ECD upon treatment with PMA for 24 h. The extracts and media were analysed by ELISA as above. (**C**) MDCK+CA IX cells were maintained overnight in serum-free medium and then treated for 2 h with PMA. Activation of CA IX shedding was revealed by immunoprecipitation–immunoblotting of the corresponding aliquots of the cell extracts and media similarly as in Figure 1. (**D**) MDCK+CA IX cells were serum-starved and then treated with PMA and/or pervanadate (V_2_O_7_) for 2 h. BB-94 was added 30 min before the induction. Collected media and cell extracts were assayed in ELISA and the results were illustrated as a ratio between the normalised absorbance data obtained from treated and control samples. (**E**) CGL3 cells grown on glass coverslips were serum-starved and then treated with PMA and pervanadate for 2 h. After allowing detachment of cells in Ca^2+^-free PBS, the coverslips were labelled with M75 followed by FITC-conjugated anti-mouse antibodies and the cell nuclei were stained with propidium iodide. The arrows indicate CA IX-positive ‘cell traces’. ^*^P<0.01 and ^**^P<0.001 by Student's *t*-test.

Shedding of cell-surface proteins is often accelerated via a signal transduction triggered by phorbol esters (such as PMA), pervanadate (V_2_O_7_) or serum growth factors. Depending on a type of the stimulus, shedding machinery involves either protein kinase C (PKC)-related or -unrelated pathways (Arribas *et al*, 1996). In order to find out whether CA IX ECD release can be regulated, we examined the effects of serum, EGF, PMA and pervanadate on the cell-bound and extracellular level of CA IX. To analyse the CA IX shedding in the presence and absence of serum, we first starved the cells in serum-free medium for 24 h and then added fresh medium containing either 10% FCS or no FCS for additional 24 h. As shown in Figure 4A, serum did not affect the release of CA IX ECD in any of the examined cell lines that naturally express CA IX, except SiHa, where it increased the cell-associated fraction of CA IX but not the ECD shedding. The lack of effect was also observed in MDCK+CA IX cells incubated in the presence or absence of either serum or EGF alone. In contrast, PMA was able to increase the level of CA IX ECD produced in CGL3, SiHa and HT-29 cells throughout 24 h (Figure 4B). A strong induction of CA IX shedding by PMA was observed in the transfected MDCK+CA IX cells already after 2 h (Figure 4C). Even higher production of CA IX ECD was induced by pervanadate, and the addition of PMA to pervanadate led to further increase (Figure 4D). Induced shedding could be inhibited by BB-94, suggesting a metalloprotease involvement also in the regulated cleavage of CA IX ECD. It is worth noting that both PMA and pervanadate stimulated also a formation of the ‘cell traces’, as revealed by the more intense immunofluorescence signal found on the glass coverslips after stripping off the treated CGL3 cells (Figure 4E). This finding supported the idea that the traces resulted from the shedding process similar to the release of CA IX ECD to a medium above the culture.

### Relationship between CA IX ECD release and expression of metalloproteases

**Figure 5:**
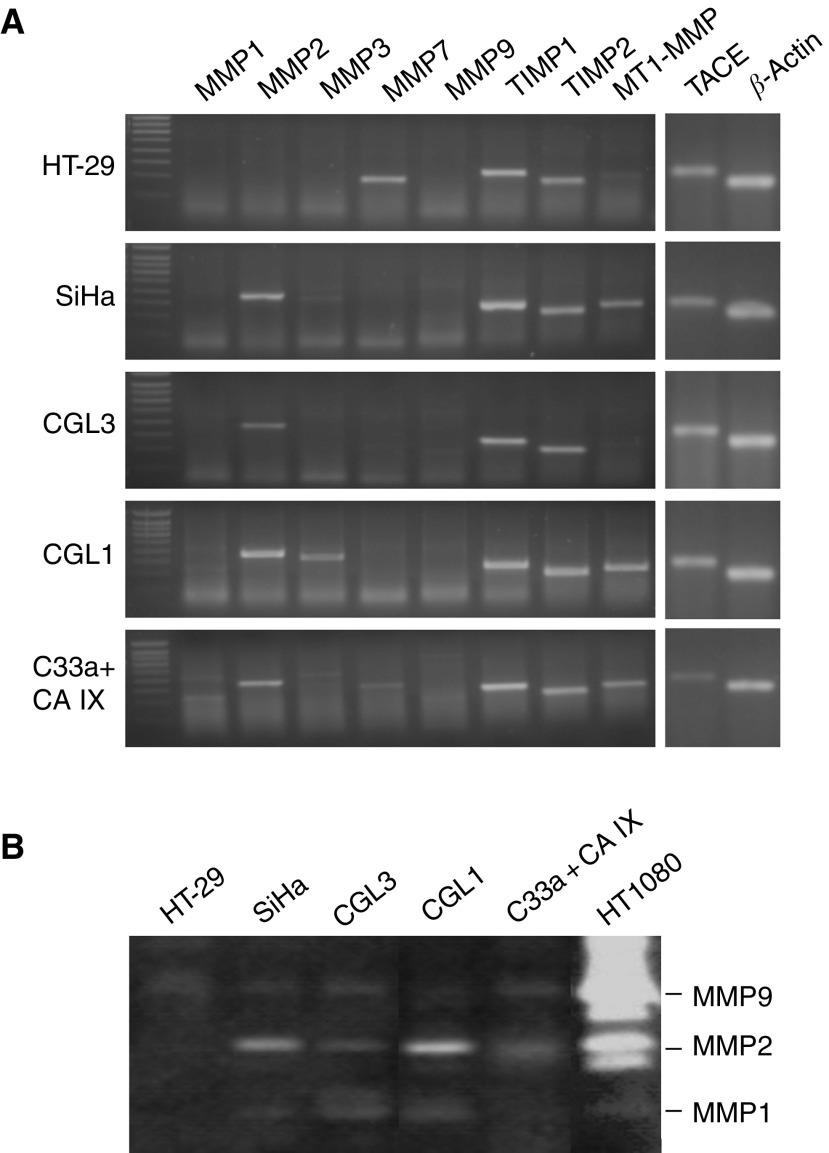
Expression of metalloproteases in selected human tumour cell lines and related collagenase activities. The cells were grown in serum-free media for 48 h. The media were collected for a collagen zymography and the cells were utilised as a source of RNA for RT–PCR. (**A**) Expression of metalloproteases was analysed by RT–PCR using the gene-specific primers listed in Table 1. *β*-Actin was used as a standard to monitor the amount of RNA templates. (**B**) Media were applied to a collagen-containing SDS–PAGE and collagenase activities of MMP1, -2 and -9 were evaluated according to an intensity of the nonstained zones representing a digested collagen. Medium from HT1080 fibrosarcoma cells was used as a positive control rich for MMP2 and -9.

Although the shedding machinery involves several common components, different cell-surface proteins can be cleaved by different metalloproteases. The enzyme–substrate relationship is determined in part by a spectrum of metalloproteases available in the particular cell/tissue type. Therefore, we were interested to look at the repertoire of metalloproteases, which are known to mediate shedding of important regulatory proteins, in our human cell models. RT–PCR analysis revealed that MMP1 and MMP9 are not expressed in any of the five cell lines that shed CA IX ECD. MMP 2, 3, 7 and MT1-MMP were absent from at least one of these cell lines (Figure 5A). In accord, the cell lines lacking expression of MMP1, -2 and -9 also failed to show the activity of the corresponding collagenases in zymography (Figure 5B). This fact disqualified the above-mentioned enzymes as potential governors of CA IX shedding. On the other hand, TACE (ADAM17) was the only examined metalloprotease whose expression could be detected in all cell lines that produce CA IX ECD (Figure 2A and C). Since TACE is well known as a molecule implicated in the shedding of many proteins, we decided to follow this clue in our further experiments.

### CA IX ECD shedding in CHO-M2 cells

**Figure 6:**
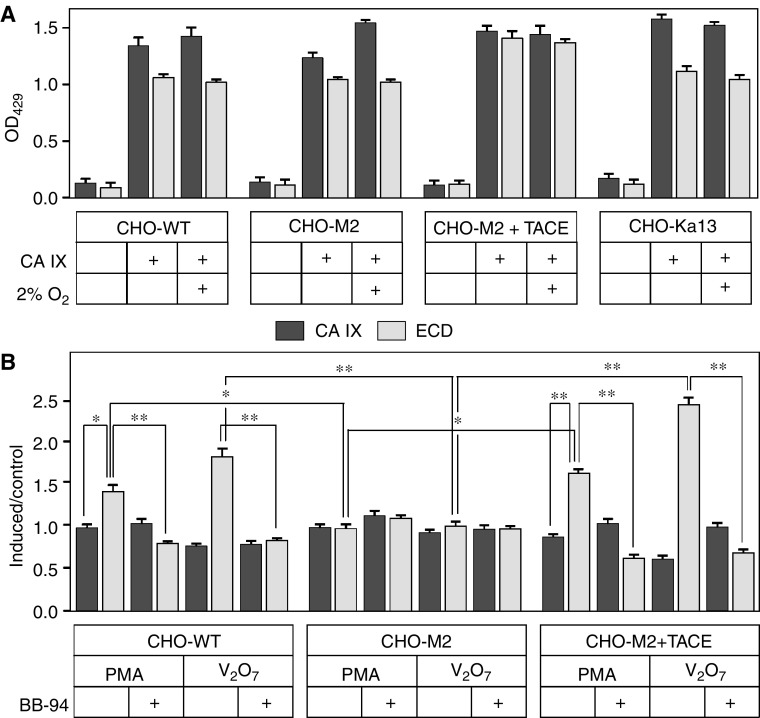
Role of TACE/ADAM17 in the basal and regulated cleavage of carbonic anhydrase IX (CA IX) ECD. CHO-wild type (WT) cells, their mutated variant CHO-M2 defective in TACE/ADAM17 maturation, CHO-M2+TACE transfectants containing a fully functional TACE and CHO-Ka13 cells lacking HIF-1*α* were transiently transfected to express CA IX. (**A**) The transfected cells were allowed to shed CA IX into the medium over 24-h incubation period under normoxia and hypoxia (2% O_2_). The same procedure was applied to mock-transfected cells. Media and extract collected from all dishes were assayed by ELISA. The results were expressed as average absorbance values with standard deviations. (**B**) The transfected cells were treated for 2 h either with PMA or with pervanadate in the absence and presence of BB-94 metalloprotease inhibitor added 30 min before the treatment. The results were illustrated as a ratio between the absorbance data obtained from the induced *vs* control samples. ^*^P<0.01 and ^**^P<0.001 by Student's *t*-test.

To evaluate a possible role of TACE in the cleavage of CA IX ECD, we examined the CA IX shedding in CHO-M2 cells that are defective in many TACE-mediated shedding events due to inactivating mutations causing its accumulation in the early secretory pathway (Villanueva de la Torre *et al*, 2004). Parental wild-type CHO-WT cells and CHO-M2+TACE transfectants expressing a functional TACE were used for comparison. In addition, we also employed CHO-Ka13 cells deficient in HIF-1*α* (Wood *et al*, 1998). All cell lines were plated to form semiconfluent monolayers and transiently transfected with a pSG5C-CA9 plasmid. Next day, the transfected monolayers were thoroughly washed, fresh medium was added and the cells were subjected to normoxia or hypoxia for 24 h. Sandwich ELISA revealed that the transfection resulted in very similar expression levels of CA IX in all cell lines. In addition, basal shedding of CA IX ECD was comparable in WT, M2 and Ka13 cells and was a little higher in the cells with reconstituted TACE (Figure 6A).

In some cases described earlier in the literature, TACE was not critical for the constitutive shedding, but was required for its activation. Therefore, we also analysed the level of CA IX ECD produced by the transfected CHO-WT, CHO-M2 and CHO-M2+TACE cells treated with PMA or pervanadate. We found that CHO-M2 cells were unable to activate cleavage of CA IX ECD, whereas CHO-WT and CHO-M2+TACE showed significantly increased shedding with both inducing factors, which was considerably reduced by BB-94 (Figure 6B). This observation strongly suggested that the regulated CA IX shedding is a TACE-dependent process.

## DISCUSSION

In the present study, we brought the evidence that the transmembrane carbonic anhydrase isoform CA IX, which is predominantly expressed in tumour cells in response to hypoxia, is shed to extracellular space in a metalloprotease-dependent manner. On this basis, CA IX can be classified among those regulatory proteins whose abundance on the cell surface and/or in the cell microenvironment is controlled via a metalloprotease-mediated ectodomain release, such as c-ErbB2, c-ErbB4, pro-EGF, CD44, c-Met, E-cadherin and others (Vecchi *et al*, 1998; Codony-Servat *et al*, 1999; Noe *et al*, 2000; Kajita *et al*, 2001; Nath *et al*, 2001; Le Gall *et al*, 2003). Basal shedding of CA IX ECD is rather inefficient, accounting for approximately 10% of the total CA IX (corresponding to about 20% of the membrane-bound protein, data not shown) as determined by ELISA in several tumour cell lines with natural expression of CA IX as well as in the transfected cells that constitutively produce ectopic CA IX. However, this is not unusual since a similarly low or even lower basal rate of ECD release was described for c-Erb2, E-cadherin and c-Met (Codony-Servat *et al*, 1999; Noe *et al*, 2000; Nath *et al*, 2001). These molecules elicit their function principally via signalling across the cell membrane and therefore it seems logical that the plasma membrane is their primary site of residence.

Hypoxia increases the expression of CA IX to a variable degree depending on a cell type. Interestingly, the cells with a low normoxic level of CA IX (CGL1 and SiHa) generally show high induction, whereas the cells with high normoxic level (CGL3 and HT-29) are less responsive. It appears that there exists certain maximum of CA IX achievable by the hypoxic induction that might be useful or required for a proper cell functioning in low oxygen conditions, namely for the control of intra- and extracellular pH and modulation of cell adhesion (Svastova *et al*, 2003, 2004). Shedding of CA IX in hypoxia generally follows an increase in total protein levels, so that the proportion of ECD relative to cellular CA IX remains the same. This may indicate that hypoxic cells have to cleave an increased number of the substrate CA IX molecules and it is well possible that a level or activity of a relevant ‘sheddase’ is enhanced by hypoxia. As a result, hypoxia simultaneously raises the cellular and extracellular content of CA IX while maintaining the normal basal rate of shedding. Although the biological consequences of the elevated amount of CA IX ECD produced from hypoxic tumour cells are unknown, this fact may have a practical value. Assessment of CA IX ECD in serum could provide information on the presence of hypoxic tumours and might be of a prognostic relevance. Data supporting this assumption are still missing; nevertheless, the recently published paper of Zavada *et al* (2003) demonstrating elevated CA IX ECD in the serum of patients with renal cell carcinomas, which frequently upregulate CA IX due to inactivating mutation of VHL tumour suppressor gene and constitutive activation of HIF-1, suggests that the idea is meaningful.

Basal rate of CA IX ECD release is not affected by serum growth factors, but can be greatly enhanced by PMA and pervanadate. This effect is clearly visible in the transfected cells that express CA IX from the constitutive promoter that allows for elimination of the transcriptional responses to inducing factors. Therefore, any PMA- or pervanadate-triggered change in the level of extracellular CA IX can be attributed to ECD shedding efficiency. Induction of shedding is much stronger in permanently transfected MDCK-CA IX cells compared to transiently transfected CHO-WT, possibly due to a higher susceptibility of MDCK cells to treatment and/or better activation of the phosphorylation signalling. Indeed, such cell-type-related differences can also be found in several studies cited herein. It is worth noting that CA IX ECD cleavage is more induced in the cells treated with pervanadate, a potent protein-tyrosine phosphatase inhibitor, than in the cells incubated in the presence of PMA, which is known to stimulate PKC. The addition of PMA can increase the pervanadate-activated release of CA IX ECD pointing out to parallel pathways that appear to involve transmission of the phosphorylation signals and encompass PKC as one of active components. This type of activation distinguishes CA IX ECD cleavage from some other cases of regulated shedding, such as c-ErB2, whose cleavage is induced by pervanadate and not by PMA (Codony-Servat *et al*, 1999), or pro-EGF and c-ErbB4 that are increasingly shed in response to PMA, pervanadate as well as to withdrawal or addition of growth factors (Vecchi *et al*, 1998; Le Gall *et al*, 2003). Although the precise molecular mechanism of regulated CA IX shedding has not been investigated so far, these initial data indicate that certain physiological situations associated with signalling events may require either quick removal of CA IX from the cell surface or rapid release of higher amount of CA IX ECD to the extracellular milieu. In this respect, it is worth mentioning that both PMA and pervanadate stimulate the shedding of CA IX ECD from the ventral side of cells to their adhesive support, which results in the formation of intense ‘cell traces’ that are very similar to traces formed by the proteolytic release of *α*_V_*β*_3_-integrin from the stressed tumour cells (Majda *et al*, 1994). This finding of a so far unclear significance is not incompatible with the proposed role of CA IX in cell adhesion (Zavada *et al*, 2000). However, better understanding of this phenomenon requires further experimental efforts.

Involvement of metalloprotease(s) in the basal shedding of CA IX has been suggested by a capacity of hydroxamate-based broad-spectrum metalloprotease inhibitor BB-94 to block this process under both normoxia and hypoxia in a concentration-dependent manner and in different cell lines. In addition, BB-94 can also inhibit activated CA IX shedding triggered by both PMA and pervanadate, indicating that it is regulated by metalloproteases responsive to both PKC-dependent and -independent signals. The putative ‘sheddase’ responsible for the basal CA IX ECD cleavage still awaits identification, since our attempts to make a deduction from the expression pattern of the most obvious candidates did not lead to any clear resolution. However, from our experiments performed with mutant CHO-M2 cells defective in shedding of many unrelated cell surface molecules due to inactivating mutation and impaired biogenesis of TACE/ADAM17 (Li and Fan, 2004; Villanueva de la Torre *et al*, 2004), it appears that TACE is not the main regulator/executor of the basal CA IX shedding. Nevertheless, it is possible that TACE can contribute to the constitutive cleavage of CA IX, because the ECD shedding in CHO-M2-TACE-transfected cells that express a functional TACE was somewhat higher than in TACE-defective CHO-M2 cells. Indeed, CHO-M2 cells are unable to activate CA IX ECD release in response to stimulation by PMA and pervanadate, whereas this activation is restored in TACE-transfected CHO-M2 cells, so TACE is clearly a proteolytic component of the regulated CA IX shedding. This feature of CA IX is reminiscent of *β*-amyloid protein, L selectin, IL-6 receptor, pro-TGF*α* and TNF*α* that all need TACE for activated release of their extracellular portions (Arribas *et al*, 1996; Li and Fan, 2004; Villanueva de la Torre *et al*, 2004). Taking these facts together, liberation of CA IX ectodomain appears to include at least two distinct metalloproteases, which play a role in different situations that may require either a constant supply of CA IX ECD or its fast and high input to extracellular space.

Irrespective of the molecular mechanism(s) behind the CA IX shedding, it is now clear that CA IX ECD is not just a remnant of unscheduled degradation, but it is a product of an active and regulated process driven by metalloproteases. At this moment, it is difficult to anticipate a biological function of the released ECD. The literature offers various examples from activated growth factors through receptor ligands toward soluble receptors regulating cellular processes via autocrine or paracrine effects (Werb and Yan, 1998). Thinking in terms of CA IX expression pattern and its proposed roles in tumour cells (Svastova *et al*, 2003, 2004; Robertson *et al*, 2004), it is likely that CA IX ECD may either interfere or cooperate with the full-length protein expressed on the cell surface, presumably by means of interactions with its binding partners. One obvious possibility would be a competitive binding of CA IX ECD to extracellular portion of anion exchanger molecules to modulate their bicarbonate transport efficiency and thereby affect the regulation of intracellular and extracellular pH in tumours, depending on actual physiological conditions and complex status of signal transduction. Although a recent finding of a direct interaction of anion exchangers with the full-length CA IX protein as well as with its engineered extracellular portion similar to ECD supports this idea (Morgan *et al*, 2005), additional extensive investigation is needed to shed light on the functional implications of the CA IX shedding and then devise strategies for its manipulation potentially leading to reduced tumour growth.

## Figures and Tables

**Table 1:** List of the primers used for RT–PCR analysis of the metalloprotease expression in the human tumour cell lines

	**Sense primer**		**Antisense primer**	
**mRNA (GenBank accession no.)**	**Position**	**Sequence (5′ → 3′)**	**Position**	**Sequence (5′ → 3′)**
MMP1 (NM_002421)	1293–1314	gatccaggttatcccaaaatga	1538–1517	caggaaaacaccttctttggac
MMP2 (NM_004530)	1002–1023	tgttcaatggcaaggagtacaa	1358–1339	ccaggaaagtgaaggggaag
MMP3 (NM_002422)	213–236	gtttgttaggagaaaggacagtgg	539–516	atatcagcctctccttcatacagc
MMP7 (BC003635)	196–217	agaagccaaactcaaggagatg	411–390	cgatccactgtaatatgcggta
MMP9 (NM_004994)	896–917	atcttccaaggccaatcctact	1082–1063	ccaggaaagtgaaggggaag
TIMP1 (BC007097)	11–32	gcgtggacatttatcctctagc	263–242	aaggtggtctggttgacttctg
TIMP2 (NM_003255)	2318–2339	atgggggttaggataggaagaa	2517–2496	ccgctgaatagaacaggctaag
MT1-MMP (X83535)	1249–1270	gcaaattcgtcttcttcaaagg	1476–1455	tgttcttggggtactcgctatc
TACE (NM_003183)	1612–1633	tggatgaaggagaagagtgtga	1874–1852	aagatccaagcaaacagtgtcat
*β*-Actin (NM_001101)	414–433	ccaaccgcgagaagatgacc	649–629	gatcttcatgaggtagtcagt
